# Lessons from first generation biofuels and implications for the sustainability appraisal of second generation biofuels^☆^

**DOI:** 10.1016/j.enpol.2013.08.033

**Published:** 2013-12

**Authors:** Alison Mohr, Sujatha Raman

**Affiliations:** Institute for Science and Society (ISS), School of Sociology and Social Policy, Law and Social Sciences Building, University Park, University of Nottingham, Nottingham NG7 2RD, UK

**Keywords:** Biofuels, Sustainability, Whole system

## Abstract

**Aims:**

The emergence of second generation (2G) biofuels is widely seen as a sustainable response to the increasing controversy surrounding the first generation (1G). Yet, sustainability credentials of 2G biofuels are also being questioned. Drawing on work in Science and Technology Studies, we argue that controversies help focus attention on key, often value-related questions that need to be posed to address broader societal concerns. This paper examines lessons drawn from the 1G controversy to assess implications for the sustainability appraisal of 2G biofuels.

**Scope:**

We present an overview of key 1G sustainability challenges, assess their relevance for 2G, and highlight the challenges for policy in managing the transition. We address limitations of existing sustainability assessments by exploring where challenges might emerge across the whole system of bioenergy and the wider context of the social system in which bioenergy research and policy are done.

**Conclusions:**

Key lessons arising from 1G are potentially relevant to the sustainability appraisal of 2G biofuels depending on the particular circumstances or conditions under which 2G is introduced. We conclude that sustainability challenges commonly categorised as either economic, environmental or social are, in reality, more complexly interconnected (so that an artificial separation of these categories is problematic).

## Highlights

• Controversy surrounding 1G biofuels is relevant to sustainability appraisal of 2G.
• Challenges for policy in managing the transition to 2G biofuels are highlighted.
• A key lesson is that sustainability challenges are complexly interconnected.

## 1 Introduction

The story of biofuels has been described as one of ‘riches to rags’ (Sengers et al., 2010). Initially cornucopian views of the potential of biofuels have been challenged under the weight of increasing speculation that their pace of development was racing ahead of understanding of the range of direct and indirect sustainability impacts of this technology. UK and EU targets for renewable fuels in the transport sector have further compounded perceptions of an unfettered dash for biofuels. Media headlines linking the rise of vast biofuel plantations in various parts of the world with rising food prices provoked a rapid shift in thinking about this technology in the second half of the 2000s. No longer is it possible to encounter the term ‘energy crops’ without some awareness of the potential conflict with the use of agricultural land for food encapsulated by the term ‘food vs. fuel’. Other social, environmental, economic and ethical challenges are emerging especially with respect to so-called ‘first generation’ biofuels produced from food crops.

Biofuels have been roughly classified to distinguish between first generation (1G) biofuels produced primarily from foods crops such as grains, sugar cane and vegetable oils and second generation (2G) biofuels produced from cellulosic energy crops such as miscanthus and SRC willow, agricultural forestry residues or co-products such as wheat straw and woody biomass. Opposition to 1G biofuels is generally assumed to be about conflict with food security. Second generation biofuels are widely seen as a sustainable response to the increasing controversy surrounding 1G, and thus distinct from it. Indeed, it has been suggested that 2G biofuels raise few ethical or sustainability issues (e.g., Charles et al., 2007; Nuffield Council on Bioethics, 2011). But will the emergence of 2G biofuels dispel claims of ‘food vs. fuel’ conflicts and what new challenges might they raise? As the world’s first commercial-scale cellulosic ethanol plant in Crescentino, Italy began operating at the end of 2012, this question is particularly timely.

## 2 Aims and methods

Examining the lessons arising from the controversy surrounding 1G biofuels, this paper assesses their relevance for perceptions of sustainability of 2G biofuels and considers the policy challenges for managing the transition to a sustainable UK bioenergy system, with particular emphasis on lignocellulosic options for biofuels. In doing so, we build on work suggesting that the ubiquitous reference to ‘food vs. fuel’ conflicts does not adequately capture the challenges posed by 1G biofuels (Raman and Mohr, in press). If this is the case, the case for 2G biofuels likewise needs to address a wider range of issues than conflict with food security alone. We draw on our experience as social scientists embedded in a major UK scientific programme on 2G biofuels where a key aspect of our work is to explore different stakeholder assessments of the sustainability of biofuels in the UK, in the context of a global bioenergy system.

Our map of sustainability issues arising from biofuels relies on the qualitative social research method of documents as a source of data and analysis (Bryman, 2012). We conducted a survey of articles in the field of energy research since the late 1970s, focusing on this flagship journal, supplemented by other key academic articles and reports produced by policy, professional and non-governmental organisations and the media. Treating these documents as a historical record of how debates about the sustainability of biofuels have evolved over time, we distilled the main themes, gaps or limitations and cross-cutting issues arising specifically around 1G biofuels. By comparison, there is less attention paid to 2G biofuel challenges in the documentary record, but we drew out the main themes where 2G was discussed.

We then tested and elaborated this map of challenges through semi-structured, in-depth interviews with 45 stakeholders from across the UK bioenergy ‘system’ (comprising science, industry, government and civil society, whilst recognising that some stakeholders may span more than one of these domains) to explore the state-of-the-art and future development of liquid transport biofuels in a global bioenergy system; and from a 2012 UK workshop involving 20 stakeholders that examined uncertainties inherent in life cycle assessment (LCA) of bioenergy and in estimations of the role of bioenergy in modelling the future UK energy mix (henceforth referenced as ‘Modelling Uncertainties Workshop’). For the interviews, the established qualitative research approach of purposive sampling was used to sample stakeholders in a strategic yet sequential way, whereby an initial sample of stakeholders was selected by virtue of their relevance to the research questions posed, and the sample gradually added to as the investigation evolved (Bryman, 2012). This allowed a variety of stakeholder assessments from across the spectrum of the UK bioenergy system to be captured.

While our analysis focuses mainly on the UK context, since national and EU biofuel targets rely, implicitly or explicitly, on imports of biomass or biofuel rather than domestic supply, we refer to global issues where appropriate. Accordingly, the key challenges for policy that we pose are UK-focused, but may have broader relevance.

Our analysis draws on work in Science and Technology Studies (STS) (Rip, 1986; Cambrosio and Limoges, 1991; Romijn and Caniëls, 2011) that argues that controversies fulfil an important technology assessment function in that they help articulate potential issues and problems that need to be considered in implementing new technologies. Irrespective of the validity of specific claims, controversies focus attention on key, often value-related, questions that were previously unrecognised and that need to be posed to address broader societal concerns. In line with Romijn and Caniëls (2011) who consider contestation and conflict as constitutive rather than constrictive of innovation systems, we suggest that controversies help to open up and expose the different elements of the socio-technical system or network which constitute a specific technology. Thus the controversy surrounding the development of particularly 1G biofuels has focused attention on the critical relationship between biofuels and sustainability that is shaping the limits of social acceptability of 2G biofuels.

The need for biofuel sustainability assessments to take into account the ‘whole system’ in an integrated manner is now generally recognised in numerous articles published in this journal and others such as *Energy*, and *Renewable and Sustainable Energy Reviews*. However, only a few of these focus specifically on lignocellulosic options for biofuels (e.g., Black et al., 2011; Haughton et al., 2009; Singh et al., 2010). The state-of-the-art of whole system assessment of biofuels is also limited in a number of significant ways.

First, the social dimension is weakly integrated (if it is considered at all) into sustainability assessments which typically focus on LCA. Yet, from an overarching whole system perspective, there is a need to put these technical assessments in the broader context of social judgments that shape views on what is considered important and why. While some key publications do consider the social dimension, they also leave some gaps. Thornley et al. (2009) focus on constraints on UK biomass supply for bioenergy, whereas a whole system analysis needs to consider the role of imports in UK bioenergy policy and sustainability issues related to biomass conversion. The sustainability framework of Elghali et al. (2007) aims to take account of different stakeholder judgments but, as they observe, the method of ranking and weighing these through multi-criteria decision analysis (MCDA) is contested. Haughton et al. (2009) incorporate stakeholder views in their sustainability assessment framework; however, theirs is a case study of the biodiversity impacts of perennial crops in two specific regions in the UK while our assessment aims to examine a range of sustainability challenges for 2G biofuels (as a whole system from field to fuel) by drawing attention to the interface between the social dimension and the mainly environmental challenges of 1G and the potential implications for 2G.

Second, most sustainability assessments used in government policy (e.g., the 2012 UK Bioenergy Strategy) and in wider debate around biofuels focus on biomass supply to the relative exclusion of issues arising from the rest of the bioenergy chain (biomass pre-treatment and conversion through to bioenergy distribution and end-use). Consequently, although issues such as energy balance across the chain are usually considered in LCA, they are not widely discussed. In this respect, the whole system of bioenergy is not really considered, nor is the wider context of the social and policy system in which bioenergy research and policy are done. Our paper fills a gap in terms of bringing the sustainability of the bioenergy whole chain to bear on social judgments around biofuels.

Opening up the black-box of controversy surrounding 1G biofuels enables us to highlight a range of emerging challenges – encompassing the social, economic, ethical, ecological and political – that threaten to compromise perceptions of sustainability of 2G biofuels. The following section draws out and critically examines the key lessons that can be drawn from the controversy surrounding 1G biofuels, assesses their relevance for 2G, and highlights the key policy challenges in managing the transition to a sustainable UK bioenergy system. The key lessons arise from the most prominent themes that emerged from the documentary and stakeholder data and focus attention on the underexplored social dimensions in these areas. Thus we do not aim to map all the relevant sustainability challenges for biofuels – this has already been attempted by other authors (e.g., Markeviĉius et al., 2010; Thornley and Gilbert, 2013).

## 3 The relevance for 2G biofuels of sustainability challenges arising from 1G

Our analysis suggests that sustainability issues identified in relation to 1G are potentially relevant to 2G, and *may* become more prominent should 2G technologies be commercialised. In part this is due to some blurring of food and non-food biomass in current and future practice which is viewed positively by some (co-products of 1G crops and fuels can be 2G bioenergy feedstocks or used for animal feed) and negatively by others (intensification of agriculture implicates the production of residues as well as the crop). Our analysis suggests the need for a more comprehensive and integrated sustainability appraisal as the challenges are more complex than implied by the ubiquitous reference to ‘food vs. fuel’ conflicts. The implications of the retrospective analysis of 1G biofuels for the sustainability appraisal of 2G biofuels, including the challenges for policy in managing the transition, are summed up in Table 1 and further elaborated in the discussion below.

### 3.1 Food security

As early as 1991, Hall (1991: 733) noted that the food vs. fuel issue is ‘far more complex than has been presented in the past and one which needs careful examination, since agricultural and export policies and the politicisation of food availability are greater determining factors’. However, the problem received scant policy attention until the 2007–08 world food price crisis that prompted warnings of sustained high food prices over the next decade as food production and supplies are displaced by biofuel production, particularly affecting developing countries that are net food importers (OECD/FAO, 2007). More recently, the negative impact of 1G biofuels on food security has been disputed (Pilgrim and Harvey, 2010) and the Nuffield Council on Bioethics (2011) notes that for every report or statement of a causal link between the 2007-08 spikes and biofuels, others provide rebuttals. However, the existence of multiple pressures on food prices such as rising meat consumption in the developing world may not exonerate biofuels. Searchinger (2011) argues ‘if it is hard to meet rising food demands, it must be harder to meet demands for both food and biofuels’.

In the 2G case, depending on the type of land used and the suitability of this land for food crops, lignocellulosic energy crops potentially constitute a conflict with food production. In theory, bioethanol from 2G agricultural residues such as wheat straw should be exempt from such a conflict. In this standard account, there is a clear distinction between using land for human food consumption versus energy production. Bringing animal feed into the equation however reveals a more complex story. On the one hand, straw may be part of the animal feed mix thus representing a link to the food chain. This link may be an important consideration for barley and oat straw which, in the UK, are added as a source of roughage in livestock feed, but wheat-straw has less feed-value in this regard (Copeland and Turley, 2008). Practices may, however, differ in other agricultural systems in other countries and hence, this issue should be checked before it is certified free of conflicts with food. Some scientists and stakeholders setting out a case for lower meat consumption argue that reserving wheat straw (and land) for biofuels is still a better use than for animal feed, a key underlying value conflict in this debate that is beginning to emerge (Centre for Alternative Technology, 2010; Carbon Cycles and Sinks Network, 2011).

#### 3.1.1 Policy challenges

‘Food vs. fuel’ could be a distorting simplification of the sustainability challenges raised by biofuels and one which overlooks the intrinsic interdependency of food and fuel; ergo, fuel is needed to produce food (Karp and Richter, 2011). Agricultural production of energy can complement food production by preventing or ameliorating rises in fuel and fertiliser prices that affect the food sector, suggest Murphy et al. (2011). But the efficacy of re-using marketable by-products and residues of bioethanol production relative to other methods for improving soil fertility should be considered (Singh et al., 2010). While acknowledging that food and fuel imperatives can conflict, Murphy et al. (2011) argue that we need better land management policies in order to reconcile and promote synergies between different uses of land for food and fuel.

The food–fuel conflict and the 1G/2G boundary are further complicated by conflicting value judgments over existing agricultural and land management practices as a whole. Using distillers’ residues from ethanol production for animal feed may be credited as good waste management in appraising the use of a food crop for 1G fuel; however, this may be judged against the sustainability of the animal food and feed industry with some arguing it may be better to use land currently used for grain fed to animals, or indeed for grazing animals, for biofuel instead (Centre for Alternative Technology, 2010). The use of land for grazing or for grain fed to animals as opposed to direct human consumption could also be independently assessed (Wassenaar and Kay, 2008). Thus the use of land for fuel needs to be considered within a broader assessment of land use for different purposes, and how land is valued (Gamborg et al., 2012).

In addition to assessments of existing agricultural management practices, policy-makers also need to consider broader (industrial, residential and recreational, etc.) land use and management practices as a whole. Yet, debates on the ‘sustainability of agriculture’ and ‘sustainability of land use’ are lacking, even within the bioenergy community. While the possibility of such a broader debate is fraught by competing social, economic, environmental and policy arguments (and the implicit as well as explicit values embedded therein) it might entail, we would suggest that, given these complexities, there is a need for such a debate.

### 3.2 Large-scale land acquisition

Linked to sustaining domestic food and energy security is the issue of land acquisition, especially on a large scale, both across and within national borders – and the uneven sustainability impacts this generates. Land in the global South acquired to produce biomass for fuel used in the global North or in domestic urban populations has been attracting critical scrutiny. The impacts of energy crop farming in developing countries have been argued to be both beneficial and harmful. Energy crops may provide income for the rural poor and lessen domestic dependency on fossil fuel imports while increasing opportunities for export revenue. But Doornbosch and Steenblik (2007) have questioned the ecological credentials of biofuels, asking ‘Is the cure worse than the disease?’ and highlighting local environmental harms to soil, water and biodiversity. NGOs have drawn attention to ‘land grabs’ leading to dispossession of local people and loss of livelihoods (Friends of the Earth, 2007; Oxfam, 2007).

Land officially designated as marginal or degraded, but suitable for 2G feedstocks, may still be relied on to fulfil the livelihood, food and fuel needs of the rural poor. For agricultural residues, the sustainable use of land in which the crop is grown may be relevant, especially as such products are increasingly seen as global commodities and traded across national borders. Biofuel companies may also target land that promises higher returns on their investments such as agricultural or irrigated land where yields are higher (science stakeholder interview, 26 October 2011; civil society stakeholder interview, 20 December 2011), or forested land where additional revenues can be gained from logging (van der Horst and Vermeylen, 2011). The intensification of agriculture raises widespread concerns of negative ecological impacts including acidification, eutrophication, ecotoxicity and ozone depletion linked to deforestation and habitat loss (Doornbosch and Steenblik, 2007; Quadrelli and Peterson, 2007; Tomei and Upham, 2009).

#### 3.2.1 Policy challenges

Given these problems, a key challenge for policy is to develop a framework for governing the practice of land acquisition in the global South. But in countries where social and environmental governance is weak, reliable monitoring of land acquisition is likely to be difficult given major power disparities between large companies and local people (van der Horst and Vermeylen, 2011). Relative priorities for different uses of land (food, fuel, grazing, recreation, biodiversity, etc.) are also likely to differ regionally or globally according to different communal or cultural value sets and need to be considered in all parts of the world (Thornley et al., 2009). Future land use priorities may also be shaped by the uneven distribution of global climate change impacts resulting in more or less emphasis placed on food or fuel production depending on local growing conditions.

### 3.3 GHG balance

Depending on where they are grown, the land management practices, modes of transportation and processing techniques used, balancing the life cycle GHG emissions remains a challenge for 2G biofuels. In the case of straw, its removal is generally associated with negative impacts on soil nutrients and structure. While returning ash to the soil after combustion could compensate for these, the lost organic matter would affect the soil organic carbon content (Thornley et al., 2009). The processing of co-products such as wheat straw also poses significant sustainability challenges in that considerable energy is required to overcome the recalcitrance of lignocellulosic biomass through pre-treatment for enzymatic saccharification (Zhu and Pan, 2009). Techniques being explored to convert agricultural co-products, woody biomass or perennial crops are therefore also attracting increasing scrutiny. The conversion inefficiency of biomass for liquid biofuels has been raised by Clift and Mulugetta (2007) who make the point that higher efficiency and better GHG savings are possible for bio-heat or combined heat and power since biomass can be directly burned rather than converted to a liquid, a step that requires further energy inputs.

If 2G biomass were sourced by felling of forests, or where the use of marginal land which is in fact a source of food stimulates indirect land use change (iLUC) effects, then the GHG balance may be questioned. A detailed analysis of the effect of iLUC by Havlík et al. (2010), paying particular attention to the issues of deforestation, irrigation water use, and crop price increases due to expanding biofuel acreage, found that 2G biofuel production powered by sustainably sourced wood (rather than fossil fuels) would reduce overall emissions but that biomass feedstocks and land use may affect other sustainability criteria like biodiversity conservation, erosion protection, or even fuelwood supply for local communities.

Yet tools used to calculate GHG emissions vary according to scope, system boundaries and data sets that can lead to large differences in the results, as shown by Whittaker et al. (2013) in a study of UK feed wheat where GHG emissions from fertiliser manufacture and application are accounted for differently and where land use change (LUC) is excluded or incompletely calculated. To conform to sustainability criteria set by regulatory frameworks such as the EU’s Renewable Energy Directive (RED), biofuel producers may select a tool that generates the greatest GHG savings.

#### 3.3.1 Policy challenges

Whether to include iLUC in GHG balance calculations is controversial. Given the complexity and uncertainty surrounding iLUC and its relevance to agriculture as a whole, deforestation (and the conversion of other forms of natural terrestrial ecosystems) may be more reliably addressed through dedicated policies that remove perverse incentives for biofuel production and reduce deforestation (wherever it occurs) through the development of strategies for sustaining forests and protecting biodiversity, rather than inclusion in GHG calculations for biofuels (Zilberman and Hochman, 2010). Assessing the energy balance of 2G biofuel production is also a central part of environmental appraisal. Here, there is some value in making the tacit assumptions and system boundaries underlying these calculations more explicit and reflecting on policy inferences from particular studies that may be more or less valid (Singh et al., 2010). This process may further blur the assumed distinction between 1G and 2G biofuels, but provides a more reasoned basis for preferring particular energy options for transport over others.

### 3.4 Environmental impacts

Biodiversity and water preservation are seen as ‘grand challenges’ that are likely to be intensified by the increasing demand for biofuels for transportation, with far-reaching social ramifications often at the local and regional scale. As early as 1991, David O Hall noted that monocultures for biofuels need to be reduced or avoided in order to maintain watersheds and ensure biodiversity. Similar concerns about monoculture plantations, especially in the global South, are echoed in recent critiques by NGOs (e.g., Action Aid, 2010).

Water is vital for maximising agricultural yield of crops grown for biomass and their residues. A study of the land and water implications of global 1G biofuel production in 2030 concluded that where traditional agricultural production already faces severe water limitations (such as in India and China, two of the world’s largest agricultural producers and consumers), strain on water resources at the local and regional level would be substantial and policy-makers would be hesitant to pursue biofuel options based on traditional food and oil crops (De Fraiture et al., 2008). Where traditional food crops such as wheat need to be grown intensively if they are to meet the various demands of food, fuel, feed and fibre, Sinclair et al. (2004) have noted that yield and water are closely correlated, thus high yielding varieties will use proportionately more water. For this reason, agricultural wastes and residues are not immune from concerns about intensive water consumption.

To avoid land conflicts with food crops, marginal and degraded land could in theory be used for dedicated energy crops to be converted into 2G biofuels, however maintaining high yields over time is dependent upon continuous access to adequate water resources (Sims et al., 2010). Arable land formerly under the EU set-aside scheme to help preserve agricultural ecosystems may, if planted with monoculture energy crops, suffer a reduction in biodiversity (RCEP, 2000). Perennial energy crops can improve biodiversity and water quality due to the reduced requirement for nitrogen fertiliser and pesticide inputs but there is serious concern about the amounts of water needed by slower growing energy crops and the possible implications for stream flow and groundwater recharge (Karp et al., 2009).

The water intensity of biomass conversion, particularly lignocellulosic conversion to biofuel, is less well known than that of biomass cultivation. However, it has been suggested that methods of recycling water for re-use in processing systems, such as for evaporative cooling, are becoming increasingly sophisticated in modern ethanol plants (IEA, 2010). Lignocellulosic conversion can also produce waste streams that can be potentially harmful to water quality and the environment (science stakeholder interview, 2 August 2011). Concerns over the direct and indirect impacts of 2G biofuels on biodiversity and water conservation therefore warrant further investigation.

#### 3.4.1 Policy challenges

While attention has been paid to methods of recycling water for re-use in biofuel processing, whole system water usage needs to be investigated (ideally across agricultural systems as a whole). Research on energy crop breed varieties that do not compromise ecosystem services is ongoing in the UK, but differences between performance in laboratory conditions and ecological conditions in situ will need to be considered, especially as impacts will be felt most keenly at the local level. Efforts to balance ecosystem services to preserve biodiversity are not new in UK agricultural systems (science stakeholder interview, 15 July 2011), however LUC impacts linked to dedicated energy crops grown in the UK and abroad will add to policy complexity (science stakeholder interview, 5 October 2011). Biodiversity and water impacts, while of global concern, tend to be location-specific, so the distribution of risks brought about by iLUC, for example, needs to be considered in policy-making in the context of uncertainties that constrain impact assessments, including the difficulty in differentiating between geospatial regions, recognising different types of water and measuring evapotranspiration (Jefferies et al., 2012).

### 3.5 Other local impacts

With the exception of GHG balance which is a global challenge unaffected by where emissions are produced or saved (Thornley and Gilbert, 2013), all the other impacts discussed so far can be described as ‘grand challenges’ whose impacts are experienced at a local level but where far-reaching, indirect social ramifications may also be felt. Human geographers highlight the necessarily spatially uneven character of sustainable transitions: that is, disproportionate (social, economic, environmental) burdens are placed on some social groups, places or ecologies, while sustainability elsewhere might be enhanced (Swyngedouw, 2007). Many of the direct impacts of biofuel production are likewise experienced at the local level especially where biomass is cultivated in poorer Southern regions for biofuel use elsewhere (van der Horst and Vermeylen, 2011).

There are widespread concerns that intensification, whether of 1G or 2G energy crops, will have negative ecological impacts including deforestation, habitat loss and declining soil fertility which in turn affect rural livelihoods. In their study of a developing biofuels sector based on Jatropha in Tanzania, van Eijck and Romijn (2008) recommend the development of policies to enhance the participation and capabilities of local communities in rural energy projects to ensure that sufficient attention is paid to their needs and preferences. Their study presents a cautionary tale for 2G that warns against a biofuels sector dominated by big commercial players interested in consolidating smaller holdings into larger plantations that will direct energy and financial revenues away from local communities. Any short-term profits offered to local farmers to sell their land to large investors will be seen as inadequate compensation for the long-term loss of livelihoods and local food and energy production.

Impacts on local communities are also a concern in the UK. Research on public attitudes to bioenergy in the UK has reported public concern about emissions and odours from bioenergy plants as well as the aesthetic impact on local landscapes (Barker and Riddington, 2003). Increased employment and financial returns to local farmers growing the biomass feedstock were seen as particular benefits of locally-sited bioenergy plants, although some concerns were expressed about the impact of heavy transport in the local area. These concerns remain relevant for 2G where, for example, the visual impact of biomass such as tall-growing miscanthus on the landscape may also be a factor depending on the location (Haughton et al., 2009). Although the higher density of woody biomass significantly reduces the need for transportation, thus limiting harmful emissions.

#### 3.5.1 Policy challenges

Siting decisions for biofuel production facilities need to consider these local impacts. Social science research has shown that public resistance to biomass development ‘in their area’ (Barker and Riddington, 2003) may be encountered, in particular where the public has not been properly consulted about the siting of a renewable energy development (Upham, 2009). A study of bioenergy developments in the Yorkshire and Humber region by Upham et al. (2007) concludes that pro-active exploration of public/stakeholder attitudes and involvement may contribute to more strategic renewable energy planning at the local level. Yet, Devine-Wright (2008) notes that social scientific scrutiny into public opinion of renewable energy technologies, local resistance or acceptance, and the ways in which public engagement with these technologies is constructed and practised in the UK, is currently limited. Thus social science has a role in highlighting that renewable energy developments are situated in places – not ‘sites’ – that involve personal, local and cultural meanings and emotions as well as physical and material properties.

## 4 Gaps and limitations in existing stakeholder appraisals that have potential implications for 2G biofuels

Drawing on stakeholder assessments identified in both the documentary review and stakeholder interviews, we call attention to a number of salient gaps and limitations in these appraisals of 1G biofuels that have potentially significant implications for the sustainability appraisal of 2G biofuels. Genetic modification and antimicrobial resistance are potentially important issues that have been neglected or somewhat marginalised in debates around biofuels, and may need to be considered. While the emerging impacts around these specific issues are difficult to quantify, they signify the importance of putting the appraisal of specific technologies in the broader context of alternative technology choices.

### 4.1 Genetic modification

Genetic modification (GM) techniques are seen by some as vital to achieving higher yields thereby increasing the net energy balance from energy crops through, for example, boosting resistance to pesticides and herbicides and to drought. The environmental impacts (both perceived and documented) of genetically modified organisms mean that GM techniques, even if they promise increased yields and a reduction in the need for crop protection, remain controversial in Europe (Gross, 2011). Yet controversy around GM may be the result of the socio-economic and political choices made rather than the technology in isolation (Levidow and Carr, 2009). However, among UK policy-makers there is currently little appetite to debate the use of GM in biofuel production while biofuels remain embroiled in debates over their broader sustainability credentials (policy stakeholder interview, 6 December 2011). The role of GM techniques in the reconstruction of 2G biofuels has received little attention (for an exception, see Levidow and Paul, 2008) by comparison with the role that synthetic biology might play in future generations, an option that has been covered widely in the media. This may yet become more widely debated if some 2G biofuels relying on GM for the development of energy crops and advanced processing techniques with improved yields become a reality. There is also the potential for going beyond the entrenched pro/anti GM debate by examining alternative uses of advanced genetics such as ‘marker-assisted’ plant breeding techniques (Stirling, 2013).

### 4.2 Antimicrobial resistance

Risk of antimicrobial resistance from the use of antibiotics in fermentation of ethanol has recently been highlighted within the scientific community (Muthaiyan et al., 2011). Where antibiotics are routinely used to control contaminants in bioethanol plants, antibiotic resistant bacteria may limit the effectiveness of antibiotics to treat future bacterial contamination. The by-products of grain processing for ethanol production, known as DDGS, contribute substantially to the economic viability of ethanol manufacturing. DDGS is increasingly used as an animal feed substitute for whole corn and soy and its potential application in other industries such as bioplastics renders it key to the long-term economic viability of the bioethanol industry. Since 2005 the EU has legislated against the use of animal feed products containing antibiotics residues as these can be directly harmful to cattle. Indirectly there may be a risk to human health if antibiotics enter the food chain through the consumption of crops fertilised with antibiotic laden manure or through absorption of antibiotics-contaminated water discharge from ethanol plants. There are concerns that the overuse of antibiotic agents in non-human settings in turn reduces the efficacy of antibiotics important for human medicine, as the antibiotics used are identical or nearly so (IATP, 2009).

## 5 Cross-cutting challenges for the whole system sustainability appraisal of 2G biofuels

Table 1 focuses on specific, commonly understood sustainability challenges but highlights the complex interconnectedness of the economic, environmental and social dimensions of these. Our analysis suggests that there are also important cross-cutting issues that emerge between the lines, or are sometimes stated explicitly in documents or interviews but that have tended to be ignored in a focus on sustainability ‘issues’, which we now summarise. Although beyond the scope of the present study, the opportunities and challenges for the whole system sustainability appraisal of 2G biofuels represented by these cross-cutting issues require further investigation in their own right.

### 5.1 Space and scale

A significant difference in the underlying framing assumptions of different stakeholder communities relates to the scale of biomass cultivation/sourcing and biofuel production. Civil society NGO stakeholders (interview, 20 December 2011; interview 20 February 2012) tend to be critical of large-scale (1G) biofuel developments due to concerns about intensification, monocultures, and human rights violations and difficulties of reliably monitoring complex North/South global supply chains and their local (spatial) impacts on sustainability (c.f. van der Horst and Vermeylen, 2011). Given that several policy scenarios (e.g., Bioenergy Strategy 2012; DECC Carbon Plan 2011) assume a key role for feedstock imports, including in the case of 2G, this is likely to remain a key issue. To highlight these problems, NGOs have tended to prefer the term ‘agrofuels’ rather than ‘biofuels’ (e.g., Action Aid, 2010). The environmental NGO, Journey to Forever (http://journeytoforever.org) argues that ‘objections to biofuels as agrofuels are really just objections to industrialised agriculture itself, along with “free trade” (free of regulation) and all the other trappings of the global food system that help to make it so destructive’. By contrast, ‘scaling up’ to large industrial production units is seen by biofuel promoters to be essential for commercial reasons, but also one of the main challenges for 2G. In principle, a more inclusive dialogue on getting the logistics right (see Sanderson (2011) on concerns currently raised by biofuel experts on the need to locate biofuel production facilities close to the point of biomass cultivation) may help mediate some of this conflict, but this also needs to be supported by consideration of the social and political tensions raised by biomass as a global commodity. Thus policy and governance mechanisms for developing a bioenergy system at different scales and that are sensitive to different spatial impacts need to be examined.

### 5.2 Trade and energy security

The initial case for 2G bioenergy rests on domestic (UK) feedstocks; in principle, this might be more stable, open to national control and less subject to international volatility once supply chains are established. In practice, biomass is an internationally traded commodity affected by a global market and WTO rules (policy stakeholder interview, 31 October 2011). Reliance on imported biomass is seen to be necessary in the short-term to meet UK and EU sustainable biofuels targets; however, even where there is a desire to change this practice, contractual terms may result in longer-term lock-in (industry stakeholder interview, 31 October 2011), an issue that would remain relevant for 2G feedstocks where these too are globally traded. Sustainability questions currently raised in connection to 1G are therefore likely to become relevant for 2G, for example, over competing uses of ‘marginal’ land for non-food energy crops or the use of residues by subsistence farmers (IEA, 2010). Where 2G feedstock trade is a more feasible option for some countries where there is no infrastructure for indigenous production, transferral of iLUC impacts to grower countries may occur. This raises the question of how trade in biomass versus trade in biofuel affects energy security concerns.

### 5.3 Environmental impact modelling

Recognition of the uncertainties, conditions and limits of the results from environmental modelling when making policy judgments and decisions on their basis was seen as crucial by stakeholders in the Modelling Uncertainties Workshop that we organised to explore issues around the interpretation of life cycle assessment (LCA) and bioenergy models. Stakeholders noted significant differences between attributional and consequential LCA^1^ in terms of levels of uncertainty, system boundaries and methodology. Yet the two approaches are sometimes conflated in the policy sphere (the EU’s Renewable Energy Directive and the UK’s Renewable Transport Fuel Obligation tend not to distinguish between the two), which can lead to the misuse of the data in policy analysis. Thus Stirling (2003) argues for the need to examine the assumptions made and boundaries drawn in quantitative environmental assessments. For example, the degree of potential reduction in GHG emissions by biofuels is dependent upon the biofuel feedstock, the management practices used and perhaps the nature (scale and distribution) of the industry (Schubert, 2006). Thus, rather than being intrinsic to the ‘renewable’ resource of biomass, environmental impacts depend also on a number of socio-technical practices. Scharlemann and Laurance (2008) use ‘total’ environmental impact assessments of different biofuel systems (considering potential losses of forests and farmland as well as GHG savings) to argue that some biofuels fare worse than fossil fuels. Stirling (2003) also argues that it is impossible to have a single standard of relative importance of impacts. The specific form of environmental impacts associated with different energy generating technologies, or even different food and fuel production processes, may be radically different. A key point that Stirling makes, which we echo, is the tendency to focus on impacts at the energy supply stage rather than in terms of subsequent use. For example, what will the relative impacts be of different transport energy choices on future air quality? Studies indicate that while combustion of renewable fuels may, in some cases, result in a reduction in regulated pollutants (e.g., CO_2_), the emissions may contain significant amounts of currently unregulated yet equally important pollutants (Gaffney and Marley, 2009).

## 6 Conclusion

The controversy surrounding 1G biofuels has fulfilled an important technology assessment function (Rip, 1986; Cambrosio and Limoges, 1991; Romijn and Caniëls, 2011) in that is has helped to articulate sustainability issues and challenges that need to be considered in implementing 2G biofuels. By drawing on the key lessons arising from 1G, we find that these are potentially relevant to the sustainability appraisal of 2G biofuels depending on the particular circumstances or conditions under which 2G is introduced. In doing so, we have highlighted the limitations of focusing on narrow framings or understandings of core sustainability challenges, such as the now ubiquitous ‘food vs. fuel’ conflict. Thus ‘food vs. fuel’ is a simplification of a complex array of interrelated factors not least to do with how land is valued, managed and governed.

A substantive lesson that we draw from opening up the different elements of the socio-technical system or network which constitutes (1G or 2G) biofuels, is acknowledging and understanding that challenges commonly categorised as the ‘three pillars’ of sustainability – economic, environmental, social – are in reality more complexly interconnected so that their artificial separation in sustainability appraisal is problematic. This point is vividly made in a study of the potential of growing perennial biomass crops, specifically SRC willow and miscanthus, for energy in the UK. Having described the potential benefits for energy security and climate policy, Karp et al. (2009) point out that these crops are physically different from currently grown arable crops – their harvesting patterns vary, they are very tall and dense, and have deeper roots, all of which has implications for a number of factors including the appearance of the rural landscape, tourist income, farm income, hydrology and biodiversity. The interrelationship of productive uses of land with the ecosystems, livelihoods and culture of specific locations, challenges notions that such connections can be simply erased and remade without cost or conflict and this has been evident in countries like the UK as well as in the global South.

At the beginning of this paper we argued the state-of-the-art of whole system assessment of biofuels was significantly limited by a tendency to focus on biomass supply to the relative exclusion of issues arising from the rest of the bioenergy chain, and by the weak integration, if at all, of the social dimension. The findings we have presented, culminating in the point made by Karp et al. (2009) above, demonstrate the importance for policy of considering the sustainability of the bioenergy whole chain in the broader context of social judgments around biofuels. To this end, we agree with Gibson (2006) who argues that an integrative understanding of sustainability appraisal calls for new forms of knowledge. Rather than treating sustainability as a matter of balancing or trading off different systems, such an approach would examine the interdependence of environmental, economic and social variables – the ‘whole system’. While we cannot claim yet to have breached the disciplinary barriers, we have begun to lay the groundwork for a more integrated sustainability appraisal.

## Figures and Tables

**Table 1:** Implications for the sustainability appraisal of 2G biofuels of challenges arising from 1G.

**Sustainability challenge**	**First generation (1G)**	**Second generation (2G)**	**Policy challenges**
**Food security**	Negative impact on food security is the biggest concern raised about using food crops and oils for producing fuel (‘food vs. fuel’).	May be relevant for non-food energy crops if grown on land having value for food production, including lignocellulose sourced from the global South.	Bioenergy can help ameliorate food price rises linked to fuel and fertiliser price rises (Murphy et al., 2011).
	Precise role of 1G biofuels in food price spikes contested.	Use of agricultural residues would not constitute a direct conflict with food (but questions may arise where biofuel production using residues is linked with 1G feedstocks).	The efficacy of re-using marketable by-products and residues of bioethanol production relative to other methods for improving soil fertility should be considered (Singh et al., 2010).
		Residues such as straw may be part of the animal feed mix and thus indirectly linked to the food chain.	Use of land for grazing or animal feed could also be independently assessed (Wassenaar and Kay, 2008).
			Sustainability of land use is a ‘wicked’ problem that extends beyond debates on the sustainability of agriculture to raise broader questions about land use and management practices as a whole.
**Large-scale land acquisition**	Where land has been acquired for sourcing biomass from global South, violations have been reported of people’s rights and livelihoods (Friends of the Earth, 2007; Oxfam, 2007).	Land officially designated as ‘marginal’ or degraded, but suitable for 2G feedstocks, may be relied on by the poor for subsistence.	Reliable monitoring of land acquisition in developing countries may be difficult given major disparities between large companies and local people (van der Horst and Vermeylen, 2011).
	LUC of natural habitats and other ecologically valuable land acquired for biofuel plantations has been linked to loss of biodiversity (Pilgrim and Harvey, 2010; civil society stakeholder interview, 6 December 2011).	For agricultural residues, the sustainable use of land in which the crop is grown may be relevant.	Relative priorities for different uses of land may differ according to different communal or cultural value sets (Thornley et al., 2009), or the uneven distribution of climate change impacts, and need to be considered in all parts of the world.
		Biofuel companies may target higher quality agricultural land (science stakeholder interview, 26 October 2011; civil society stakeholder interview, 20 December 2011) or forested land that will provide additional income from logging (van der Horst and Vermeylen, 2011), and intensification can further exacerbate ecological impacts.	
**GHG balance**	iLUC effects including the release of carbon stocks from conversion of forests, peatlands or grasslands for biofuel crops (Fargione et al., 2008) will reduce carbon savings.	If feedstocks were sourced by felling of forests, or where the use of ‘marginal’ land which is in fact a source of food stimulates iLUC effects, GHG balance may be questioned.	Given complexity of iLUC and its relevance to agriculture as a whole, deforestation may be more reliably addressed through dedicated policies rather than inclusion in GHG calculations for biofuels (Zilberman and Hochman, 2010).
	Impact of nitrogen fertilizers and energy costs of transporting feedstocks can affect net energy balance.	Soil organic carbon content is affected by removal of straw (Thornley et al., 2009).	Results of calculations depend on system boundaries and assumptions which need to be explicit to avoid misuse by decision-makers (Singh et al., 2010; Modelling Uncertainties Workshop, 2012).
	Most conventional biofuels depend on fossil fuels for their production, (House of Commons Environmental Audit Committee, 2008).	Net energy balance is relevant given the energy inputs needed to break down lignocellulosic material and for transportation of bulky residues.	
	Carbon calculators used to test GHG emissions show large differences, mainly due to how emissions from fertiliser manufacture and application are accounted for and whether LUC is excluded or incompletely calculated (Whittaker et al., 2013).	Although, cellulosic ethanol requires less fossil fuels for process heat and electricity than starch-based ethanol (AEA/NNFCC, 2010).	
		Biofuel producers may select a carbon calculator that generates the greatest GHG savings (differences in emission factors yield different results) to comply with sustainability criteria (Whittaker et al., 2013).	
**Environmental impacts**	Biodiversity and water preservation are seen as ‘grand challenges’ with far-reaching social ramifications.	Perennial energy crops can improve biodiversity and water quality due to the reduced requirement for nitrogen fertiliser and pesticide inputs; but slow-growing crops may affect groundwater recharge and require constant access to water (Karp et al., 2009).	Whole system water usage needs to be investigated (ideally across agriculture as a whole).
	Concerns about the impacts of monocultures for biofuels on biodiversity and water conservation, especially linked to the conversion of natural terrestrial ecosystems, are not new.	Biodiversity and water impacts remain a concern: high-yielding food crops grown for their co-products and residues will use disproportionately more water while marginal or degraded land (Sims et al., 2010) or land formerly under the EU set-aside scheme (RCEP, 2000) may be targeted for monoculture energy crops.	Research on energy crop breed varieties that protect ecosystem services is ongoing, but differences between performance in laboratory conditions and ecological conditions in situ will need to be considered.
		Whole chain water use a concern (especially where 2G processing techniques are particularly water intensive).	
			Efforts to balance ecosystem services to preserve biodiversity are not new in UK agricultural systems (science stakeholder interview, 15 July 2011), however LUC impacts in the UK and abroad will add to policy complexity (science stakeholder interview, 5 October 2011).
			Impacts tend to be location-specific, so distribution of risks is an issue: yet impact assessments on biodiversity and water are constrained by considerable uncertainties including geospatial differentiation, different types of water and evapotranspiration (Jefferies et al., 2012).
**Other local impacts**	Intensification of energy crops has been linked to long-term loss of livelihoods and local food/energy production through displacement of local subsistence farmers (van Eijck and Romijn, 2008) and negative ecological impacts.	The visual impact of biomass (e.g., tall-growing miscanthus) and biomass plants on the landscape may be a factor, depending on the location.	Siting decisions for biofuel production facilities need to consider these local impacts, preferably in (early) consultation with the local community.
	Biomass plants can have a negative impact on local air quality through processing and transport emissions and on the aesthetics of the local landscape.	Processing and transport emissions remain a concern.	Understanding how public perceptions are shaped by broader social, cultural and personal meanings and assessments of bioenergy developments can help to develop more robust policy decisions, and social science can help in this regard.

## Footnotes

1 LCA was originally developed to determine the direct environmental impacts of a single existing product or process (‘attributional’) but has been increasingly used to inform policy decisions around the potential in/direct impacts of future large-scale (product and process) systems (‘consequential’), such as a biorefinery (Whittaker et al., 2011). Attributional LCA focuses on describing the environmental flows to and from a product or process and is most relevant for regulatory purposes, while consequential LCA describes how environmental flows will change in response to possible policy decisions and is most relevant for policy analysis (civil society stakeholder interview, 9 August 2011; science/government stakeholder interview, 26 January 2012). Ultimately, the differences between the two are the result of the choices made in defining the goals and system boundaries of the LCA process and other methodological choices, although these are not always made transparent.
